# The protective role of perceived support, job recognition and availability of workplace resources in the relationship between burnout and suicidality among health and social care professionals in Switzerland

**DOI:** 10.3389/fpubh.2026.1839667

**Published:** 2026-06-24

**Authors:** Dolores Angela Castelli Dransart, François Delavy, François Geiser, Ramona Patt, Laurent Michaud, Stéphane Saillant, Sylvie Lapierre, Karl Andriessen, Serjara Aleman, Christian Maggiori

**Affiliations:** 1 School of Social Work Fribourg, HES-SO University of Applied Sciences and Arts Western Switzerland, Fribourg, Switzerland; 2 Psychiatric Liason Service, University Hospital (CHUV), Lausanne, Switzerland; 3 University of Lausanne, Lausanne, Switzerland; 4 Department Adult Psychiatry, Psychiatry Neuchâtel Center, Neuchâtel, Switzerland; 5 Centre for Research and Intervention on Suicide, Ethical Issues and End-of-Life Practices (CRISE), Montreal, QC, Canada; 6 Centre for Mental Health and Community Wellbeing, Melbourne School of Population and Global Health, The University of Melbourne, Melbourne VIC, Australia

**Keywords:** burnout, health-social care personnel, job recognition, suicidal ideation, suicidality, suicide, support, workplace resources

## Abstract

**Introduction:**

Health and social care professionals are at a higher risk of suicide. This study explores the mediating role of perceived private support (from family and friends), workplace support (from colleagues, supervisors and superiors), perceived availability of workplace resources and perceived job recognition in the relationship between burnout and suicidality among these professionals.

**Methods:**

A cross-sectional study was conducted using an online questionnaire distributed across 21 partner organizations, primarily in the mental health sector, spanning seven Swiss states in three linguistic regions (German, Franch, Italian). Data from 2,388 health and social care professionals (physicians, psychiatrists, psychologists, social workers and other health and social care personnels) were analyzed using double mediation structural equation modeling.

**Results:**

Burnout was significantly associated with higher levels of suicidality. It was negatively associated with perceived workplace support and private support, both of which, in turn, predicted lower suicidality. Both forms of support independently attenuated the association between burnout and suicidality. Similarly, perceived job recognition and availability of workplace resources were associated with lower suicidality and independently mitigated the association between burnout and suicidality. 42% of respondents were classified as at risk of burnout. No gender differences were found.

**Discussion:**

The findings suggest that, while suicidality is a complex issue with many contributing factors, increased organizational support could reinforce the protective role of certain work-related factors. Consistently providing adequate support and resources in the workplace, as well as enhancing job recognition, are likely to reduce burnout-associated suicidality. Organizational measures to address burnout are also warranted, since distressed professionals may compromise quality of care, patient safety and institutional functioning or even leave the profession. Therefore, beyond personal suffering, the distress of personnels should also be addressed as a public health issue, especially in times of personnel drop out and shortages.

## 1 Introduction

Suicidality (suicidal ideation and suicide attempts) as well as death by suicide, are a public health problem (1) with devastating and long-lasting impacts on individuals and communities worldwide. In recent decades, several countries have developed national suicide prevention strategies to improve the care and support for individuals at risk of suicide and their families (2). However, suicide prevention among social and healthcare professionals is often overlooked, despite some evidence suggesting that they are at a higher risk of dying by suicide (3–6) or of experiencing suicidality (7, 8). Previous studies have mainly focused on physicians and nurses- (6) as well as emergency service workers (9).

Physicians were considered an at-risk group in some meta-analysis (4, 6). Physicians in Spain had higher rates of suicide than the general population, especially female (10). In Austria, the SMRs (Standardized mortality ratio) were also higher than the general population among female physicians. Nursing professionals, especially females, were found to be at an increased risk of suicide compared to the general population (3, 11). In the USA suicide risk level for social workers was 56% higher than that of the working age population but not higher compared to professional managerial workers (12).

Healthcare professionals also report suicidal ideation or suicide attempts. The Mental Health of Nurses and Doctors (MeND) survey carried out in 29 countries (European Union, Iceland and Norway) show that 14% of physicians and 13% of nurses reports thoughts of being better off dead or of harming themselves in the 2 weeks prior to the survey (13). In Turkey, 22.3% of physicians reported wishing of being dead and 9.6% had suicidal ideation in the previous month; 8.6% had attempted suicide in their lifetime (7). In the USA, 5.5% of nurses reported suicidal ideation in the year prior to the survey (14). More than one-third (35.8%) of Spanish students in social work reported having suicidal ideation, and 9.6% had attempted suicide at some point (15).

Healthcare professionals are also likely to be affected by burnout. Burnout has been defined as such: “In a worker, occupational burnout or occupational physical AND emotional exhaustion state is an exhaustion due to prolonged exposure to work-related problems” (16). Healthcare experience burnout in various dimensions such as emotional exhaustion, cynicism or depersonalization (negative or excessive detached attitudes), and a lack of personal accomplishment (feeling of incompetence and reduced work performance) (17, 18).

Compared to the general working population, physicians (19) experience higher levels of burnout. Some studies found burnout prevalence rates ranging from 42% (20) to approximately 50% among physicians (21). A systematic review within the medical field reported great variability in the prevalences due to differences in burnout definition, assessments methods and cutoff (22).

In a study among US nurses, 38.2% of the participants exhibited at least one symptom of burnout, with 34.4% scoring highly for emotional exhaustion (14). Among mental health professionals, a meta-analysis indicated that the overall estimated pooled prevalence was 40% for emotional exhaustion, 22% for depersonalization, and 19% for low personal accomplishment (23). Another meta-analysis of prevalence reported that 50% of social workers suffered from emotional exhaustion, 45% of depersonalization and 39% of low personal accomplishment (24).

Previous studies have identified work-related stressors that are associated with burnout. These include workload, sense of control over one's activities, relationships with colleagues, perceived support from superiors, availability of workplace resources, recognition and rewards, and threats of violence from patients (18, 25).

With regard to suicidality, working conditions may contribute to 10–13% of suicide deaths (26) yet this remains an understudied area of research. General work stress (as experienced subjectively) was a significant predictor of suicidal ideation among Turkish healthcare workers, with job satisfaction having a mediating effect (27).

The presence or absence of support is also likely to influence suicidality or burnout. Studies have found that availability of social support protects against suicidality (28). Specifically, greater social support acted as a buffer for healthcare workers experiencing suicidal ideation and behaviors (29), for paramedics (30), and junior doctors (31). Correspondingly, low social support was found to be a risk factor for suicidality among Turkish physicians (7). A lack of perceived leadership support was significantly associated with greater odds of suicidal ideation among US non-physician hospital staff (32).

Support from colleagues and superiors has also been shown to mitigate the effects of high demands or workloads and negative working conditions on junior doctors (31), and to reduce emotional exhaustion among healthcare workers in Taiwan (33) and social workers (25). Supportive work environments and relationships mitigate workplace stress (34, 35).

A significant association between burnout and suicidality was found among physicians (36) and US nurses (11).

In summary, physicians, nurses and other health workers are at risk of death by suicide, a significant number experience burnout, and some suicidality. Some work-related stressors were found to be correlated with suicidality or burnout, with a direct relationship between the last two reported. Nevertheless, the mechanisms likely to explain this relationship remain unclear (37), especially with regard to protective factors. Thus, this article aims to examine the relationship between burnout and suicidality by adopting a mediation framework and asking the question: What role do some workplace factors play in the association between burnout and suicidality among health and social care professionals? The hypotheses are that: 1) perceived social support (whether private or at work) and 2) perceived job recognition and perceived availability of workplace resources partially mediate the association between burnout and suicidality.

## 2 Materials and methods

This cross-sectional study was based on a questionnaire administered to health and social care professionals in seven Swiss states (cantons). The study was conducted in collaboration with field partners from 21 organizations in three regions of Switzerland (German, French, and Italian Speaking).

### 2.1 Data collection

Data was collected between April 2023 and May 2024 via an online survey created using Qualtrics (38). A convenience sample was obtained through a call for participation sent by a contact person at each partner organization to all employees. The inclusion criteria were: employment at a partner organization; work in healthcare or social care; age > 18 years; and fluency in German, French or Italian. All professionals were explicitly invited to complete the questionnaire, regardless of their state of distress at the time of data collection, for comparison purposes. For ethical reasons, participation was voluntary. The average response rate by organization was 23%.

Participants were informed of the study's purpose, potential risks and benefits, and assured of the protection of their data, before explicitly consenting to participate. The study complied with Swiss standards and requirements for data protection and with Swiss and international ethical standards for research with human beings (39, 40). Ethical approval for this study was provided by the Ethics Committee for Research on Human Beings of the Canton of Vaud (CER-VD) (Project ID 2022-01956). Participation was anonymous, and no direct identifiers were collected. A safety protocol was implemented that included internal organizational resources for participants, where available, along with a list of external support persons (psychiatrists or psychotherapists) that they could contact if needed. The first consultation session was provided free of charge.

### 2.2 Measures

The Suicidal Ideation Scale (SIS) (41) assesses suicidality on a “continuum ranging from covert suicidal thoughts to more overt or intense ideation and ultimately, actual suicide attempts” (41) (*p*. 175). The ten items are scored on a 5-point Likert scale (1 = Never to 5 = Always). Respondents report how they have felt or behaved over the past 12 months. The SIS score is a sum ranging from 10 to 50. Higher scores correspond to more severe suicidality. Luxton et al. (42) confirmed the psychometric properties of the SIS, providing further support for its use in research. In the present study, the internal consistency was α = 0.89.

The Professional Fulfillment Index (PFI) (43) is a 16-item instrument designed to evaluate physicians' professional fulfillment and burnout over the previous 2 weeks. The Overall burnout scale comprises ten items from the PFI (α = 0.93). It combines the work exhaustion subscale (four items, α = 0.86) and the interpersonal disengagement subscale (six items, α = 0.93), each with an 11-point Likert response scale ranging from 0 (not at all) to 10 (extremely). Higher scores correspond to higher levels of burnout.

Perceived private support and workplace support were each assessed with a single item designed for the study: “During difficult times, have you been able to count on the support of your personal /workplace social environment?” Participants rated their perception of support from both their workplace and personal environment (private support) on a scale from 0 (not at all) to 10 (completely).

Perceived job recognition was assessed using a single item designed for the study: “Generally speaking, do you feel that your work is recognized for its true value within your institution?” Participants rated their perception of job recognition on a scale from 0 (not at all) to 10 (completely).

Perceived availability of workplace resources was assessed using a single item designed for the study: “Do you currently feel that you have enough resources and tools to cope with the demands and expectations in your workplace?” Participants rated their perception of available resources and tools on a scale from 0 (not at all) to 10 (completely).

Gender identity was assessed using a single item, in which participants were asked to indicate how they identify, with response options including ‘woman', ‘man', ‘other', or ‘prefer not to say'.

The single-item questions were designed with the help of professionals from institutions and selected members of the Scientific Research Board. Table 1 provides a summary of the observed variables and their roles in the structural equation models.

**Table 1:** Latent constructs, observed variables, and their roles in the structural equation models.

Model	Variable name	Variable type	Role in SEM	Item description	Measurement scale	Instrument/ Reference
1+2	Suicidality	Latent construct	Outcome	Please indicate how often you have experienced the following feelings or engaged in the following behaviors during the past 12 months: 1. I have been thinking of ways to kill myself.	Likert (1–5)	SIS (41)
				2. I have told someone I want to kill myself	Likert (1–5)	SIS (41)
				3. I believe my life will end in suicide.	Likert (1–5)	SIS (41)
				4. I have made attempts to kill myself.	Likert (1–5)	SIS (41)
				5. I feel life just isn't worth living.	Likert (1–5)	SIS (41)
				6. Life is so bad I feel like giving up.	Likert (1–5)	SIS (41)
				7. I just wish my life would end.	Likert (1–5)	SIS (41)
				8. It would be better for everyone involved if I were to die.	Likert (1–5)	SIS (41)
				9. I feel there is no solution to my problems other than taking my own life.	Likert (1–5)	SIS (41)
				10. I have come close to taking my own life.	Likert (1–5)	SIS (41)
1+2	Burnout	Latent construct	Predictor	During the past two weeks I have felt… a. A sense of dread when I think about work I have to do	Likert (0–10)	Work exhaustion (43)
				b. Physically exhausted at work	Likert (0–10)	Work exhaustion (43)
				c. Lacking in enthusiasm at work	Likert (0–10)	Work exhaustion (43)
				d. Emotionally exhausted at work	Likert (0–10)	Work exhaustion (43)
				During the past two weeks my job has contributed to me feeling… a. Less empathetic with my patients	Likert (0–10)	Interpersonal disengagement (43)
				b. Less empathetic with my colleagues	Likert (0–10)	Interpersonal disengagement (43)
				c. Less sensitive to others' feelings/emotions	Likert (0–10)	Interpersonal disengagement (43)
				d. Less interested in talking with my patients	Likert (0–10)	Interpersonal disengagement (43)
				e. Less connected with my patients	Likert (0–10)	Interpersonal disengagement (43)
				f. Less connected with my colleagues	Likert (0–10)	Interpersonal disengagement (43)
1	Perceived private support	Observed variable	Mediator	During difficult times, have you been able to count on the support of your personal social environment?	Likert (0–10), treated as continuous	Self–developed
1	Perceived workplace support	Observed variable	Mediator	During difficult times, have you been able to count on the support of your workplace social environment?	Likert (0–10), treated as continuous	Self–developed
2	Perceived workplace resources	Observed variable	Mediator	Generally speaking, do you feel that your work is recognized for its true value within your institution?	Likert (0–10), treated as continuous	Self–developed
2	Perceived job recognition	Observed variable	Mediator	Do you currently feel that you have enough resources and tools to cope with the demands and expectations in your workplace?	Likert (0–10), treated as continuous	Self–developed

### 2.3 Data analysis

The data were analyzed using R (44) and the lavaan package (45) for structural equation modeling (SEM). The SEM approach was chosen to examine the direct and indirect effects of burnout on suicidality. Two separate mediation models were fitted because the two groups of mediators concern different timeframes (for support: times of distress; for work-related factors: time of survey).

In the first model, we focused on the extent to which individuals felt they could rely on their networks in times of distress — that is, the perceived availability of support in case of distress. We distinguished between two sources of support: perceived private support, provided by close personal relationships such as partners, family members and friends, and perceived workplace support, received from colleagues, supervisors and institutional actors. We modeled both private and workplace support as parallel mediators to allow a more accurate evaluation of their protective effect and relative importance. In a second model, we investigated whether two work-related factors, namely perceived job recognition and perceived availability of workplace resources, mediate the association between burnout and suicidality.

Each model included a correlation between the two mediators to account for their potential shared variance. This acknowledges that the two forms of support and work-related factors may be related beyond their common relationship with burnout.

To achieve an acceptable model fit, we iteratively modified the measurement model by adding theoretically justifiable residual covariances between items with high modification indices. The same eleven correlated residuals were introduced to improve each model specification and account for shared variance among conceptually related items. Model fit was evaluated using a combination of fit indices: the Comparative Fit Index (CFI), Tucker-Lewis Index (TLI), Root Mean Square Error of Approximation (RMSEA), Standardized Root Mean Square Residual (SRMR), and Normed Fit Index (NFI). A model was considered to show acceptable fit if CFI, NFI and TLI were ≥0.9 (46), RMSEA ≤ 0.08 (47), and SRMR ≤ 0.08 (48). Although the likelihood ratio χ^2^ is reported, we did not use it as a primary index of model fit due to its known sensitivity to large sample sizes (49, 50).

Additionally, a multigroup analysis was conducted to determine whether gender moderates the relationships in the double mediation models. Those who self-identified as ‘Other' (*n* = 6, 0.3%) or ‘Prefer not to say' (*n* = 11, 0.5%) for gender were excluded from the multigroup analyses due to insufficient sample sizes.

### 2.4 Participants

From an initial sample of 2,816 respondents, we excluded 207 participants (7.4%) who did not work within an institutional setting and could therefore not provide institutional-related information. Additionally, only cases with complete data for all scales and items used in the analyses were retained, resulting in the removal of 221 participants (7.8%) with missing data. We opted not to impute missing values for the Suicide Ideation Scale (SIS) for three reasons: (1) the items within the scale assess distinct aspects of suicidal ideation or behavior, which have varying inter-item correlations; (2) maintaining the diversity of responses across items is clinically significant; and (3) exploratory factor analysis confirmed this heterogeneity within the scale structure. A similar approach was applied to the Overall Burnout Scale. The final sample included 2,388 participants working in healthcare and social work in Switzerland. The questionnaire was completed in French by 1,124 participants (47.1%), German by 1,061 participants (44.4%), and Italian by 203 (8.5%).

## 3 Results

Most participants (76.1%) identified as women, 23.2% as men, and 0.7% as another gender or declined to say. The average age was 41 years (SD = 10.8, range 18–68). Most participants were nurses (46.3%), followed by psychologists and/or psychotherapists (12.6%), physicians with psychiatry title (9.8%), and other types of physicians (7.3%). A few indicated working in social work (7%), and 17% in another occupation. The majority of participants (64.1%) indicated working in the field of mental health.

The mean SIS score was 12.2 (SD = 4.2, range 10–46), with 53.2% of respondents selecting ‘Never' for all items (resulting in an SIS score of 10). The mean burnout score was 3.2 (SD = 2.1, range 0–10). According to the cut-off point proposed by Trockel et al. (43), 42.9% of respondents were likely at risk of experiencing burnout. The mean level of perceived private support was 8.2 (SD = 2.1), with 6.9% of respondents scoring below 5. The mean level of perceived workplace support was 6.2 (SD = 2.8), with 24.9% of respondents scoring below 5. For perceived job recognition, the mean level was 5.1 (SD = 2.8), with 40.7% scoring below 5. For perceived availability of workplace resources, the mean level was 6.1 (SD = 2.5), with 26.5% scoring below 5.

### 3.1 Model 1–Burnout-suicidality mediated by private support and workplace support

**Figure 1:**
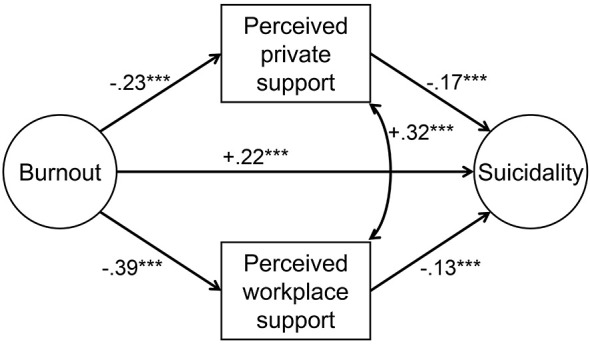
Structural equation model illustrating the associations between burnout, suicidality, perceived private support and perceived workplace support in model 1. Standardized estimates are reported. Asterisks denote significance levels: ^*^p < 0.05, ^**^p < 0.01, ^***^p < 0.001.

Model 1 showed satisfactory fit criteria (χ^2^ (196) = 2,482.8, *p* < 0.001, RMSEA = 0.07, CFI = 0.92, TLI = 0.91, SRMR = 0.06, NFI = 0.92) and a significant positive association between burnout and suicidality (see Figure 1) (standardized estimate, β = 0.22, *p* < 0.001). This relationship is partially mediated by perceived private support and perceived workplace support.

The indirect effects show that burnout is negatively associated with perceived workplace support (β = −0.39, *p* < 0.001) and perceived private support (β = −0.23, *p* < 0.001). In turn, presence of each type of support predicts lower levels of suicidality (professional support: β = −0.13, *p* < 0.001; private support: β = −0.17, *p* < 0.001). The estimated correlation between workplace support and private support was significant (*r* = 0.32, *p* < 0.001), suggesting that these two sources of support are linked even after accounting for their relationship with burnout.

To assess the extent of mediation, we first examined the total effect of burnout on suicidality without mediators (β = 0.30, *p* < 0.001). When both support variables were included as mediators in the model, the direct effect decreased to β = 0.22, *p* < 0.001, representing a 28.0% reduction in magnitude of the coefficient. This reduction, combined with the persistence of a significant direct effect, confirms partial mediation.

The model explained 14.6% of the variance in suicidality (R^2^ = 0.146). Together, the mediating effects accounted for approximately 28.5% of the variance explained in suicidality. Specifically, workplace support accounted for 16.2% of the variance and private support for 12.3%. The model also explained 14.9% of the variance in workplace support and 5.2% of the variance in private support.

In summary, while burnout shows a direct relationship with suicidality, both kinds of support act as protective factors, with each type of support independently attenuating the relationship between burnout and suicidality.

### 3.2 Model 2–Burnout-suicidality mediated by perceived job recognition and perceived availability of workplace resources

**Figure 2:**
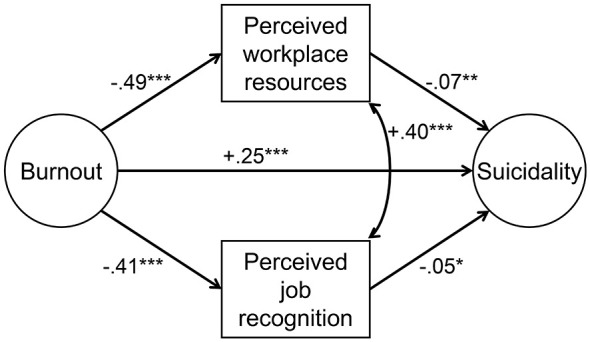
Structural equation model illustrating the associations between burnout, suicidality, perceived job recognition and perceived availability of workplace resources in model 2. Standardized estimates are reported. Asterisks denote significance levels: ^*^p < 0.05, ^**^p < 0.01, ^***^p < 0.001.

Model 2 showed satisfactory fit criteria (χ^2^ (196) = 2,650.3, *p* < 0.001, RMSEA = 0.08, CFI = 0.92, TLI = 0.9, SRMR = 0.07, NFI = 0.91) and showed a significant positive association between burnout and suicidality (see Figure 2) (standardized estimate, β = 0.25, *p* < 0.01). This relationship is partially mediated by perceived job recognition and perceived availability of workplace resources.

The indirect effects indicate that burnout is negatively associated with perceived job recognition (β = −0.41, *p* < 0.001) and perceived availability of workplace resources (β = −0.49, *p* < 0.001). In turn, their presence predicts lower suicidality levels (perceived job recognition: β = −0.05, *p* < 0.05; perceived availability of workplace resources: β = −0.07, *p* < 0.01). The estimated correlation between perceived job recognition and perceived availability of workplace resources was significant (*r* = 0.40, *p* < 0.001), indicating that these two work-related factors are associated even after accounting for their relationship with burnout.

To assess the extent of mediation, we examined the total effect of burnout on suicidality without mediators (β = 0.30, *p* < 0.001). When perceived job recognition and perceived availability of workplace resources were included as mediators in the model, this direct effect was reduced to β = 0.25, *p* < 0.01, representing a 17.2% reduction in the coefficient magnitude. This reduction, combined with the persistence of a significant direct effect, confirms that we are in the presence of a partial mediation.

The model explained 10.2% of the variance in suicidality (R^2^ = 0.102). Together, the mediating effects account for approximately 17.9% of the explained variance in suicidality. Specifically, perceived job recognition accounts for 6.7% of the variance, and perceived availability of workplace resources for 11.2%. The model also explained 16.5% of the variance in perceived job recognition and 23.9% of the variance in perceived availability of workplace resources, the mediating variables.

In summary, while burnout shows a direct relationship with suicidality, both perceived job recognition and perceived availability of workplace resources serve as protective factors, each attenuating the relationship between burnout and suicidality independently.

### 3.3 Multigroup analysis

A multigroup analysis was conducted to determine whether gender moderates the relationships in the double mediation models. To evaluate gender moderation, we tested configural, metric, and scalar invariance across men and women groups for both model 1 and 2. The models showed acceptable fit at each level of invariance, suggesting that the model structure is comparable across genders. The results in Table 2 show that fit indices met established thresholds for invariance testing (51), with negligible ΔCFI, ΔRMSEA, ΔTLI, and ΔSRMR values supporting that gender does not significantly moderate the relationships in either model.

**Table 2:** Goodness-of-fit indices for gender invariance testing across models.

Model	Invariance Level	RMSEA	CFI	TLI	SRMR	ΔRMSEA	ΔCFI	ΔTLI	ΔSRMR
Model 1	Configural	0.08	0.92	0.91	0.06	—	—	—	—
	Metric	—	—	—	—	−0.0011	−0.0013	0.0027	0.0026
	Scalar	—	—	—	—	−0.0004	−0.0001	0.001	0.0011
Model 2	Configural	0.08	0.91	0.9	0.06	—	—	—	—
	Metric	—	—	—	—	−0.0012	−0.0013	0.0031	0.0028
	Scalar	—	—	—	—	−0.0005	0.0001	0.0013	0.0008

Due to unequal group sizes and the limited number of participants in some occupational categories, multigroup analyses by profession were unfeasible. However, exploratory analyses indicated negligible associations between occupation and the study variables (R^2^ < 0.02 across outcomes).

## 4 Discussion

This article aimed to improve the understanding of the relationship between burnout and suicidality by exploring the mediating role of perceived private and workplace support, perceived availability of workplace resources, and perceived job recognition. We hypothesized that these variables would influence this relationship. Our findings support this hypothesis and are likely to contribute to the existing literature by helping to clarify the underlying processes of the associations between burnout and suicidality.

In line with the findings of previous studies (36, 52) our results indicates that there is a significant positive association between burnout and suicidality among social and healthcare professionals. However, burnout may not yet be sufficiently taken into account with regard to suicidal ideation (53). The relationship between burnout and suicidality is complex, with multiple factors likely influencing it. In the present study, the four explored variables–perceived private support, perceived workplace support, perceived availability of workplace resources, and perceived job recognition– all proved to play a role to a certain extent. Previous studies (28–30, 32) have found support to influence both suicidality and burnout. Our results build on these findings by showing that perceived private support and perceived workplace support during distressing times can mediate the relationship between burnout and suicidality. Distinguishing between private and workplace support allows our findings to highlight the specific protective aspects of each type of support. Given that workplace support plays an important role in the relationship between burnout and suicidality, increased organizational support may be warranted. Current provisions at work still leave room for improvement in the support offered: 24.9% of respondents rated workplace support below 5 out of 10. It would also be necessary to verify whether professionals perceive the support available to them as adequate, timely and whether they appreciate it. A supportive work environment has been identified as an effective coping mechanism for employees (54, 55). During the pandemic of COVID, healthcare workers in Australia expressed a desire for various forms of support, such as empathic validation of their distress, improved access to mental healthcare, and a stronger sense of belonging and connection with their peers (56). The need for compassionate leadership within healthcare organizations that offers an environment of psychological safety and supports teams in preparing for and debriefing from difficult events (54, 56) was also mentioned. Leaders who address psychosocial hazards are essential for improving the disclosure of distress and help-seeking endeavors (57). Building a supportive community can reduce isolation, burnout and stigma (57, 58). Providing support in the workplace seems even more important given that, for the participants of this study, the perception of availability of workplace resources at work also plays a mediating role in the relationship between burnout and suicidality. Improvement in this regard also seems possible, since 26.5% of our study's respondents rated the perceived workplace availability of resources below 5 out of 10.

Web applications designed to help prevent burnout and proactive offering of support among healthcare professionals could be an option, as could clinical supervision, ongoing educational opportunities, and spaces dedicated to sharing and addressing the emotional and psychological impact of care issues (11, 59). It seems appropriate to proactively offer support to professionals, since seeking help may be hindered in case of suicidal ideation. Physicians with suicidal ideation were found to be less likely to seek help than the colleagues without it (60). Peer support to foster integration and provide assistance could also help professionals (11).

Recognition at work also appears to be important for the burnout-suicidality relationship. In our study, perceived job recognition mediated the relationship between burnout and suicidality. This factor could be further fostered, given that 40.7% of our respondents rated perceived job recognition below 5 out of 10. Among healthcare workers in Turkey, job satisfaction was found to have a mediating effect between general work stress and suicidal ideation (27).

Our findings show that 42.9% of professionals might be at risk of experiencing burnout. This figure requires attention at both the individual and system levels. At the organizational level, it is necessary to promote a culture that is caring and supportive (14, 54), to provide fair work conditions and constant support in the workplace to ensure the safety of both professionals and patients. Actually, a meta-analysis found that the positive effects of organizational interventions on burnout symptoms (e.g. shortening shifts or modifying work processes) were greater than those of a range of individually focused interventions (61, 62). Without proper support, and beyond the personal impact, distressed professionals may compromise both the quality of care and patient safety which are very relevant public health issues. Furthermore, professional burnout or distress may represent a significant factor in addressing the health personnel shortage in Europe (13).

At an individual level, early detection is paramount, as well as easy, timely and non-stigmatizing access to support and care. Therefore, support provided to individuals, as well as support through improved institutional processes and culture, seem critical and necessary.

### 4.1 Limitations and strengths

This study has several limitations. Firstly, invitations to participate and recruitment were carried out through the participants' organizations. Selection bias may have occurred, and we cannot verify how diligently the contact person within each partner organization distributed the invitation to participate. Furthermore, professionals may not have trusted that data collection would be anonymous and independent, and that employers would not have access to the data. Moreover, some potential participants may have chosen not to participate due to the sensitive nature of the subject, possibly because they did not wish to confront the issue directly or because it caused them distress. It is unclear whether the situation of the respondents was similar to or different from that of the non-respondents in terms of levels of distress or suicidality.

Secondly, for ethical and organizational reasons, the study is based on self-perception and on a convenience sample with voluntary participation. Consequently, the findings cannot be generalized. Nevertheless, they can contribute to scientific knowledge and conversation by highlighting the importance of the studied variables in the association between burnout and suicidality.

Thirdly, only four variables were used to model the relationship between burnout and suicidality, despite the fact that suicidality is a complex, multifactorial issue. Other variables, such as depression, are likely to influence this relationship, as indicated by the variance explained by the models. Furthermore, using single items for the mediators may have limited measurement sensitivity, potentially weakening detectable associations. Further studies are warranted to investigate this complex relationship.

Finally, the cross-sectional design of the study only allowed data to be collected at one point in time. Longitudinal studies are needed to understand the phenomenon over time and deepen our knowledge.

Despite its limitations, this study also has important strengths. Firstly, while the sample was convenience-based, the study's value lies in its ability to collect rare and highly sensitive data among a significant number of social and health professionals.

Secondly, the analytical models are originals, and two models were distinguished: one related to factors at the time of distress and the other to work conditions at the time of data collection. The first model investigated the distinct roles of private and workplace support in the relationship between burnout and suicidality, showing that workplace support plays a dominant role. This calls for action in the workplace.

Thirdly, to our knowledge, this is the first study to investigate various vocations in several fields, including health, mental health and social care, and within various cultural, linguistic, and institutional settings. Notably, data on social care professionals was virtually non-existent in Switzerland and other European countries. Further analysis is ongoing with regard to potential specificities in various settings and vocations.

Fourthly, the global study aimed to investigate both risk and protective factors, as well as the interplay between different kinds of factors: personal and work-related.

Finally, the completely anonymous online format, which allowed participants to complete the questionnaire remotely without personal contact or exposure, and at their own pace, could have encouraged participation.

## 5 Conclusion

Results of the present study contribute to literature and scientific knowledge by highlighting the role of perceived private and workplace support, perceived availability of workplace resources and perceived job recognition in the association between burnout and suicidality amongst social and health professionals. Our findings suggest that consistently providing workplace support and sufficient workplace resources as well as improving job recognition are measures likely to mitigate burnout-related suicidality. Organizational and public health measures are therefore paramount in addition to individual support measures or support provided by family and friends.

## Data Availability

The dataset presented in this article is not readily available because of ongoing further analysis, data protection and for ethical reasons. Reasonable requests to access the dataset should be directed to Dolores Angela Castelli Dransart angela.castelli@hefr.ch.
